# Revision Stapedectomy in a Female Patient with Inner Ear Malformation

**DOI:** 10.1155/2016/8520703

**Published:** 2016-04-06

**Authors:** Tirth R. Patel, Aaron C. Moberly

**Affiliations:** The Ohio State University Wexner Medical Center, 915 Olentangy River Road, Suite 4000, Columbus, OH 43212, USA

## Abstract

*Objectives*. We describe an unusual case of surgical management of congenital mixed hearing loss in a female patient with inner ear malformation. This report outlines the role of temporal bone imaging and previous surgical history in evaluating a patient's risk of perilymph gusher during stapes surgery.* Methods*. A 68-year-old female patient with a history of profound bilateral mixed hearing loss due to ossicular and cochlear malformation presented to our otology clinic. She had undergone multiple unsuccessful previous ear surgeries. Computed tomography revealed bilateral inner ear malformations. She elected to proceed with revision stapedectomy.* Results*. The patient received modest benefit to hearing, and no operative complications occurred.* Conclusions*. Although stapedectomy has been shown to improve hearing in patients with stapes fixation, there is risk of perilymph gusher in patients with inner ear abnormalities. Evaluation and counseling of the risk of gusher during stapes surgery should be done on a case-by-case basis.

## 1. Introduction

Stapes fixation is a relatively common cause of conductive hearing loss, but it rarely presents with concomitant inner ear malformation, comprising a special challenge for the otologist. The combination of these two conditions has been well described previously in male patients with X-linked mixed hearing loss [1]. In this report, we present a unique case of a female patient with profound hearing loss due to congenital stapes fixation and inner ear malformation.

## 2. Case Presentation

A 68-year-old female patient with a history of congenital hearing loss presented to the otology clinic to discuss management of her hearing loss. She had previously been diagnosed with ossicular and cochlear malformation but had no significant family history of hearing loss. She had undergone several surgeries in the right ear between the ages of 4 and 36 years to improve her hearing. Previous surgeons had attempted this by stapedectomy and implantation of various stapes prostheses in the patient's right ear, and she reported immediate hearing improvement after the most recent surgery. However, several hours after that surgery she vomited and the hearing improvement ceased. Previous surgical records could not be obtained. At the time of the initial visit, she used behind-the-ear hearing aids bilaterally with poor benefit.

**Figure 1:**
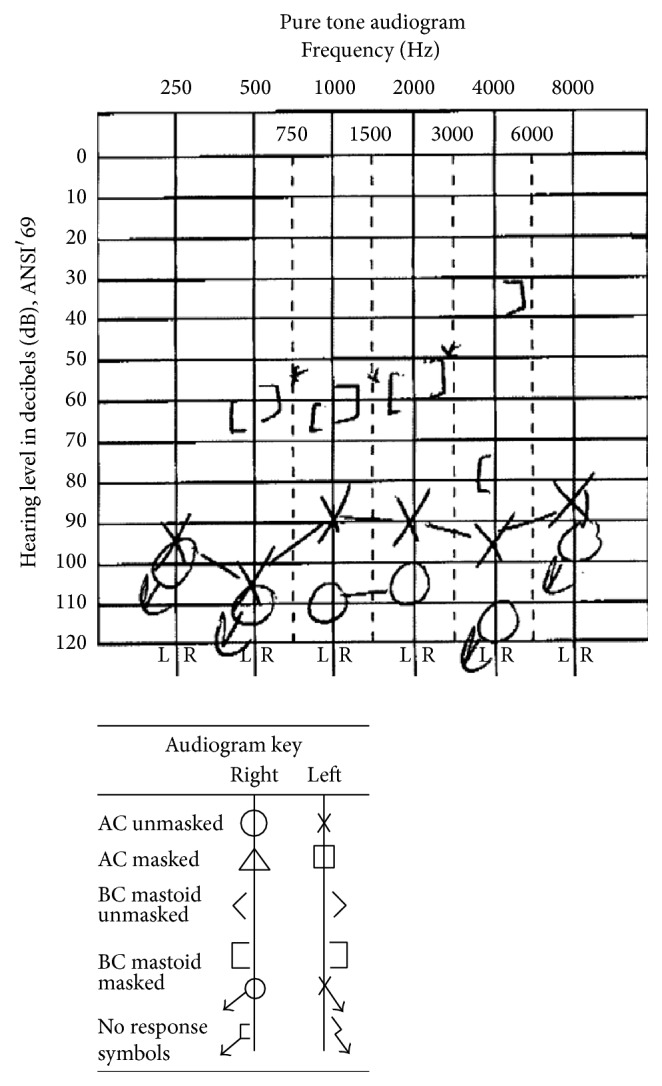
Patient's pure-tone audiogram, revealing a profound mixed hearing loss bilaterally.

A previously obtained audiogram revealed a profound mixed hearing loss bilaterally (with a masking dilemma due to large bilateral conductive components), with air-conduction pure-tone averages (500, 1000, and 2000 Hz) of 108 dB HL in the right ear and 95 dB HL in the left ear (Figure 1). Word recognition scores were 20% at 115 dB HL in the right ear and 80% at 105 dB HL in the left ear.

**Figure 2:**
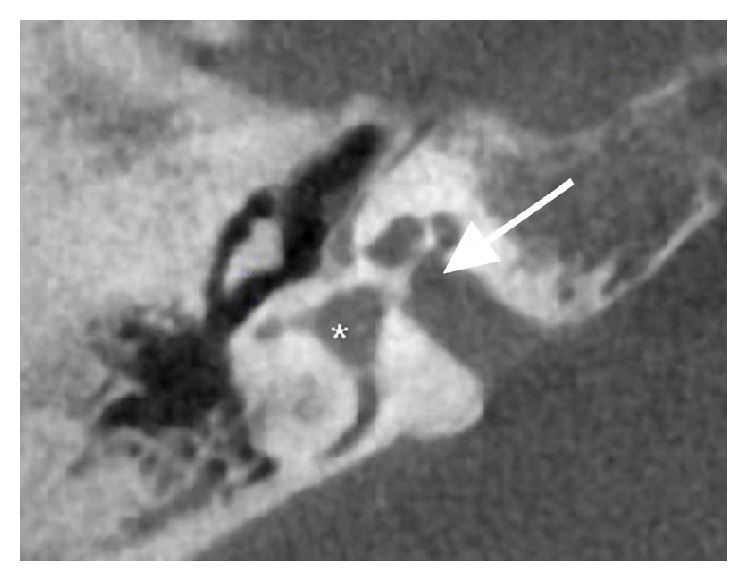
Axial noncontrast computed tomography of right temporal bone reveals incomplete separation of the right internal auditory canal from the basal turn of the cochlea (arrow) and an enlarged vestibule (asterisk).

**Figure 3:**
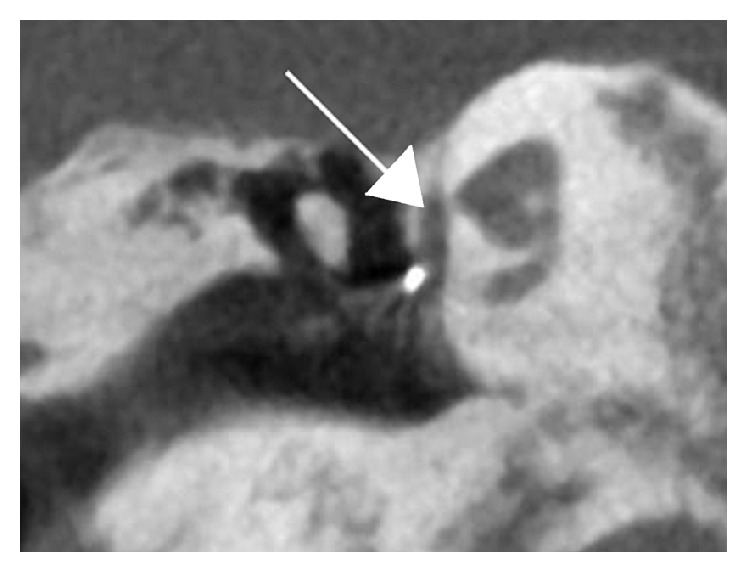
Coronal noncontrast computed tomography of right temporal bone reveals the end of a previously placed stapes prosthesis (metallic object) abutting the second genu of the facial nerve (arrow), which is descending anterior to the promontory as evidenced by its location adjacent to the turns of the cochlea.

The patient had never undergone otologic imaging. Because of the patient's unusual story and multiple previous surgeries, a noncontrast temporal bone computed tomography (CT) scan was obtained. Results revealed multiple bilateral middle ear and cochlear malformations. The ossicles demonstrated abnormal morphology and appeared to be fused bilaterally. There were only 1.5 turns to both cochleae instead of the expected 2.5 turns. The vestibules were enlarged bilaterally. Incomplete separation of the basal turns of both cochleae from the internal auditory canals (IACs) was seen (Figure 2). A metallic stapes prosthesis was seen in the right ear, with abnormal orientation, abutting the facial nerve; the bilateral facial nerves followed an abnormal course of the second genu anterior to the promontory (Figure 3).

After thorough discussion of risks, benefits, and alternatives of surgery, the patient elected to undergo a right revision stapedectomy under general anesthetic. During surgery, the incus and malleus were identified as partially fused and malformed. Two prostheses from previous surgeries were found unattached to any ossicles and were removed. A vestibulotomy was made with a KTP laser anterior to an opening from a previous operation that, on review of the CT, appeared to open into a superior extension of a hypotympanic air cell. On creating the new vestibulotomy, a pulsatile but low-flow perilymph leak was noted, which was slowed by placing a tragal perichondrium graft over the vestibulotomy. A new titanium total ossicular chain prosthesis was then placed onto the graft at the site of the vestibulotomy, which resulted in cessation of the perilymph leak. Perichondrium and tragal cartilage were used to support the tympanic membrane over the prosthesis, and absorbable gelatin foam pieces were used to pack the middle ear and stabilize the prosthesis. The external auditory canal was then filled with antibacterial ointment and a cotton ball was placed, concluding the procedure.

Three weeks after surgery, the patient returned for a postoperative visit. She was doing well and reported minimal dizziness postoperatively, improved hearing in the operated ear, and no evidence of cerebrospinal otorrhea or rhinorrhea. Three months postoperatively, she was doing well with subjective improvement in hearing and a well-healed ear. Postoperative audiogram showed a persistent right-sided mixed hearing loss, with a modestly improved unaided pure-tone average of 90 dB HL and an aided pure-tone average of 45 dB HL, although her word recognition remained at 20% unaided. Subjectively, she was happy with her outcome and improvement using her hearing aid. We discussed the possibility of cochlear implantation in the future, but she was content with her current status using hearing aids.

## 3. Discussion

The patient presented in this report was likely diagnosed with congenital stapes fixation as a child and had undergone several stapedectomy attempts in the right ear to correct it. Congenital stapes fixation presents with a conductive hearing loss early in childhood. Juvenile otosclerosis is another entity that may cause a progressive fixation of the stapes, leading to a conductive hearing loss in the pediatric patient. For these patients, however, onset of hearing loss is typically after puberty. Stapedectomy has been proven to be an effective treatment for congenital stapes fixation [2, 3]. A recent review of surgical outcomes found that the mean air-bone gap of 189 ears that underwent stapedectomy closed from approximately 40 dB HL preoperatively to 11 dB HL postoperatively [2]. Our patient had a history of several failed surgeries resulting in no hearing benefit or only short-lived benefit, likely because the vestibulotomy was not performed in the correct place or because the prostheses used did not remain in good position. Due to the above evidence that she had some previous surgical benefit without major complications, we elected to pursue a revision stapedectomy, which led to a partial closure of her air-bone gap.

A major concern, for which extensive preoperative counseling and discussion was held with the patient, was the potential for a perilymph gusher (i.e., stapes gusher) with her inner ear malformation identified on CT. Perilymph gusher is a rare complication of stapes surgery but may have a tremendous adverse effect on the patient's hearing [3]. Patients with inner ear malformation are at a much higher risk of developing a gusher during surgery [4]. Therefore, it is crucial that a temporal bone CT scan is taken prior to surgery for patients with congenital conductive hearing loss to determine the patient's risk. Findings on CT that indicate a high risk for perilymph gusher include cochlear malformation, dilated IACs, incomplete separation between the cochlea and IAC, and enlarged vestibular and cochlear aqueducts [4–6]. The CT scan of the patient presented here showed cochlear malformation along with an incomplete separation between the IACs and basal turns of the cochleae, which put her at relatively high risk for intraoperative gusher. However, the patient's history of multiple ear surgeries without known incidence of previous perilymph gusher indicated that revision stapedectomy would be a reasonable option to consider. Although a temporal bone CT is vital to assessing risk for developing a perilymph gusher, there have also been prior reports in which CT has failed to detect patients at risk [7]. As a result, it is important for the surgeon to preoperatively counsel all patients undergoing stapes surgery on the risk of a gusher and to be prepared to encounter one during surgery.

Perilymph gushers are common in individuals with X-linked mixed hearing loss. This is a well-known condition with many similarities to the case presented here. Patients present with severe mixed hearing loss in childhood due to congenital stapes fixation and inner ear malformation. A characteristic temporal bone CT will reveal bulbous IACs with only partial or no separation of the IAC from the cochlea, giving the patients a very high risk of perilymph gusher [5]. This condition is inherited in an X-linked recessive manner and occurs almost exclusively in male patients. Female carriers of one copy of a mutant gene associated with this X-linked mixed hearing loss may have normal hearing or only mild hearing loss, but they rarely have the inner ear malformations seen in affected males [8, 9]. Our female patient's history and imaging findings were consistent with those that are characteristic of X-linked mixed hearing loss; however, she did not have a family history of hearing loss. On review of the literature, only one other case of likely X-linked mixed hearing loss in a female patient has been published [9]. This entity, while very rarely seen in female patients, suggests that imaging should be recommended prior to surgical intervention for any patient with congenital conductive hearing loss, regardless of gender.
